# Efficacy and safety of evocalcet in Japanese peritoneal dialysis patients

**DOI:** 10.1007/s10157-019-01692-y

**Published:** 2019-04-06

**Authors:** Kazuhiko Tsuruya, Ryutaro Shimazaki, Masafumi Fukagawa, Tadao Akizawa, Evocalcet Study Group, Yoshimitsu Hayashi, Hidetomo Nakamoto, Shoji Koga, Ichiro Okido, Minoru Kubota, Fumihiko Koiwa, Masahiro Takeda, Terumasa Hayashi, Makoto Hiramatsu, Hideki Kawanishi, Hidetoshi Kanai, Sakuya Ito, Kazuhiko Tsuruya, Koji Mitsuiki, Hirofumi Ikeda

**Affiliations:** 1 Department of Nephrology, Nara Medical University, 840 Shijo-cho, Kashihara, Nara 634-8521 Japan; 2 R&D Division, Kyowa Hakko Kirin Co., Ltd., 1-9-2 Otemachi, Chiyoda-ku, Tokyo, 100-0004 Japan; 3 Division of Nephrology, Endocrinology and Metabolism, Department of Internal Medicine, Tokai University School of Medicine, 143 Shimokasuya, Isehara, Kanagawa 259-1193 Japan; 4 Division of Nephrology, Department of Medicine, Showa University School of Medicine, Namics 301, 4-24-51 Takanawa, Minato-ku, Tokyo, 108-0074 Japan

**Keywords:** Evocalcet, Calcimimetic, Peritoneal dialysis, Parathyroid hormone, Secondary hyperparathyroidism, Phase III study

## Abstract

**Background:**

Secondary hyperparathyroidism (SHPT) is a serious and common complication in patients receiving peritoneal dialysis (PD). Cinacalcet is currently the recommended therapy for SHPT; however, gastrointestinal (GI)-related symptoms can result in low adherence and high discontinuation rates. Evocalcet is a novel calcimimetic agent that has non-inferior efficacy while providing a more tolerable safety profile.

**Methods:**

This was a multicenter, intra-subject dose-adjustment treatment study evaluating the efficacy and safety of 1–8 mg evocalcet orally administered once daily for 32 weeks for the treatment of SHPT in PD patients. Patients then entered a 20-week extension period (dose range 1–12 mg). The primary endpoint was the proportion of patients who achieved a mean intact parathyroid hormone (iPTH) level of 60–240 pg/mL during the evaluation period (weeks 30–32). Secondary efficacy endpoints included the proportion of patients achieving ≥ 30% decrease in iPTH levels.

**Results:**

A total of 39 Japanese PD patients with SHPT received evocalcet. The target mean iPTH level of 60–240 pg/mL was achieved by 71.8% (28/39) of patients during the evaluation period and 83.3% (20/24) of patients at week 52. The proportion of patients who achieved ≥ 30% decrease in iPTH levels from baseline was 74.4% (29/39) during the evaluation period and 87.5% (21/24) at week 52. Adverse drug reactions occurred in 46.2% (18/39) of patients, with most being of mild-to-moderate severity including GI-related events.

**Conclusion:**

This study shows the long-term efficacy and safety of evocalcet when orally administered to PD patients with SHPT once daily.

**Clinical trial registration:**

ClinicalTrials.gov: NCT02549417, https://clinicaltrials.gov/ct2/show/NCT02549417; JAPIC: JapicCTI-153016, http://www.clinicaltrials.jp/user/showCteDetailE.jsp?japicId=JapicCTI-153016.

**Electronic supplementary material:**

The online version of this article (10.1007/s10157-019-01692-y) contains supplementary material, which is available to authorized users.

## Introduction

The number of patients receiving chronic dialysis therapy is increasing every year and exceeded 2 million people worldwide in 2010 [1]. Of these, the number of patients receiving peritoneal dialysis (PD) was 196,000 [2]. In Japan, there are currently over 300,000 patients receiving dialysis therapy, and approximately 9000 (2.7%) of these cases are for PD [3]. Secondary hyperparathyroidism (SHPT) is a serious complication in patients receiving hemodialysis (HD) or PD. There are no significant differences in the mechanism of onset and pathological state of SHPT between patients receiving HD and PD. Therefore, these patients receive similar treatments, including cinacalcet hydrochloride (cinacalcet), active vitamin D, and phosphate binders [4]. Because of the nature of PD, oral treatments are the preferred route of administration instead of intravenous injection. Currently, cinacalcet is the only oral calcimimetic agent approved for SHPT patients receiving PD. However, cinacalcet is associated with gastrointestinal (GI)-related symptoms as well as pharmacologic issues, such as cytochrome P450 (CYP) 3A4-mediated metabolism and CYP2D6 inhibition.

Evocalcet, a novel oral calcimimetic agent, is an allosteric modulator that acts on the calcium-sensing receptors on the membrane of parathyroid cells to suppress parathyroid hormone (PTH) secretion [5]. Compared with cinacalcet, evocalcet has non-inferior efficacy and a lower incidence of GI-related adverse events (AEs), and a clinical trial in HD patients showed it to be a more tolerable therapy in comparison [6]. In a previous phase I study, the safety, pharmacokinetics, and pharmacodynamics of a single oral dose of evocalcet in SHPT patients receiving PD (*n* = 9) was evaluated (unpublished data, NCT02143271; JapicCTI-142537). This open-label, multicenter, single-dose study showed that evocalcet effectively decreased intact PTH (iPTH) and serum calcium levels in PD patients. Additionally, because there was no drug clearance by dialysis, there were no marked differences in the plasma concentration of evocalcet and other pharmacokinetic parameters when compared with a similar previous clinical study in SHPT patients receiving HD [7, 8].

The present phase III study aimed to assess the efficacy and safety of evocalcet orally administered once daily for 32 weeks in Japanese PD patients with SHPT. The study also examined the long-term safety and efficacy of treatment with evocalcet by continuing the treatment for an additional 20 weeks after completion of the initial 32-week trial period.

## Methods

### Patients

Patients were enrolled in this phase III study based on the following inclusion criteria: age ≥ 20 years, stable chronic kidney disease with a history of PD for ≥ 16 weeks before screening, iPTH level of > 240 pg/mL at screening, and a corrected serum calcium level of ≥ 8.4 mg/dL at screening. Corrected serum calcium was calculated as follows: if serum albumin was < 4.0 g/dL, then corrected serum calcium (in mg/dL) = serum calcium (in mg/dL) + [4.0 − serum albumin (in g/dL)]; if serum albumin was ≥ 4.0 g/dL, then corrected serum calcium = serum calcium [9].

The exclusion criteria were as follows: use of cinacalcet within 2 weeks before screening; change in dose or dosing regimen of an activated vitamin D drug or its derivative, phosphate binder, or calcium preparation within 2 weeks before screening; treatment with bisphosphonates, denosumab, or teriparatide within 24 weeks before screening; and parathyroidectomy within 24 weeks before screening. Patients requiring concomitant HD or hemodiafiltration, or with changes in their dialysis condition (type of dialysis, such as continuous ambulatory PD, or automated PD; dialysate calcium level; the frequency of daily exchanges; and the volume of dialysate retained per session) within 4 weeks prior to screening were excluded.

### Study design

**Fig. 1:**
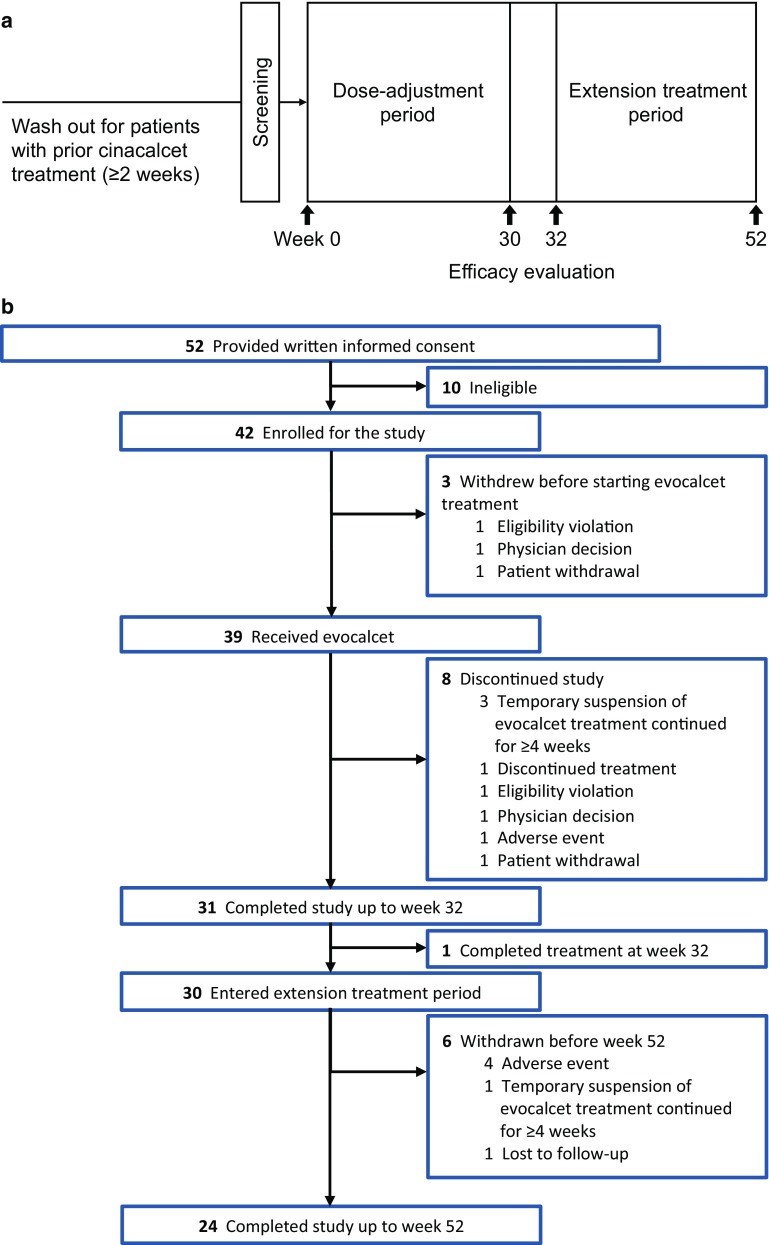
Study design (**a**) and patient flow diagram (**b**). *FAS* full analysis set

This was a phase III, multicenter, open-label, intra-subject, dose-adjustment study that aimed to determine the efficacy and safety of evocalcet orally administered once daily in PD patients with SHPT (Fig. 1a). The study consisted of a 30-week dose-adjustment period (weeks 0–30) and a 2-week evaluation period (weeks 30–32). Patients could withdraw from the study at week 32 prior to initiation of a 20-week extension period (weeks 32–52) of evocalcet orally administered once daily to evaluate the long-term safety and efficacy of evocalcet.

Patients self-administered evocalcet once daily over the treatment period with the primary aim of maintaining an iPTH concentration between 60 and 240 pg/mL based on the target range proposed by the Japanese Society for Dialysis Therapy [10]. A pill count was performed to confirm patient compliance. The starting dose was set at 1 mg evocalcet or 2 mg evocalcet if patients had an iPTH concentration of ≥ 500 pg/mL and a corrected serum calcium concentration of ≥ 9.0 mg/dL at the time of the screening.

To maintain an iPTH concentration of 60–240 pg/mL, the following dose-adjustment criteria determined whether the evocalcet dosage was increased or decreased during the treatment period: the dosage of evocalcet was increased to a maximum of 8 mg once daily during the dose-adjustment period and to a maximum of 12 mg during the extension treatment period by increments of 1 mg if the current dosage was maintained for at least 4 weeks, the iPTH concentration was > 240 pg/mL, the corrected serum calcium level was ≥ 8.4 mg/dL, or the investigator deemed a dose escalation likely to be safe. During the evaluation period, the dosage at the end of the dose-adjustment period was maintained. The dose of evocalcet was reduced in 1-mg increments if the iPTH concentration was < 60 pg/mL or if an AE that was determined to warrant an evocalcet dose reduction was present.

Treatment could also be temporarily interrupted for a maximum of 4 weeks if the corrected serum calcium level was ≤ 7.5 mg/dL or if the investigators judged that treatment should be interrupted based on safety concerns for the patient. Resumption of interrupted treatment was based on the corrected serum calcium levels increasing to ≥ 8.4 mg/dL.

The concomitant use of cinacalcet, bisphosphonates, denosumab, and teriparatide was prohibited throughout the study. Changes in medications, such as active vitamin D preparations and their derivatives, phosphate binders, and calcium preparations, were permitted (type, dosage, and mode of administration) from start of treatment through to week 52. Dialysis type, frequency, and dialysate volume retained could also be changed after the study commenced. However, dialysate calcium levels could not be changed until week 32, but could be altered during the extension period.

### Biochemical determinations

All biochemical tests were performed in central LSI Medience Corporation (Tokyo, Japan) laboratories. Intact PTH was measured using electrochemiluminescence immunoassay (ECLusys PTH; Roche Diagnostics K.K., Tokyo, Japan). Intact fibroblast growth factor 23 (FGF23) was measured using enzyme-linked immunosorbent assay (FGF-23 ELISA Kit; KAINOS Laboratories, Inc., Tokyo, Japan). All biochemical determination tests were performed before administration of evocalcet on scheduled visits.

### Efficacy analysis

The primary efficacy endpoint was the number and percentage of patients who achieved a mean iPTH concentration of 60–240 pg/mL during the evaluation period (weeks 30–32). The mean iPTH concentration was determined at weeks 30 and 32. The secondary efficacy endpoints included the mean percentage change in iPTH concentration during the evaluation period from baseline, and the number and percentage of patients who achieved a ≥ 30% decrease in iPTH concentration during the evaluation period from baseline.

The mean ± standard deviation (SD) iPTH concentration (pg/mL), corrected serum calcium concentration (mg/dL), serum phosphorus concentration (mg/dL), whole PTH concentration (pg/mL), ionized calcium concentration (mEq/L), intact FGF23 concentration (pg/mL), corrected serum calcium-phosphorus product (mg^2^/dL^2^), bone metabolism markers (bone-specific alkaline phosphatase [BAP], tartrate-resistant acid phosphatase 5b [TRACP-5b], and total procollagen type 1 intact N-terminal propeptide [P1NP]) were also assessed.

### Safety analysis

Safety endpoints included recording AEs, drug-related AEs, GI-related AEs, clinical laboratory values (e.g., corrected serum calcium concentrations), body weight, vital signs, and 12-lead electrocardiogram (ECG). All tests were performed before administration of evocalcet on the visit day, except for the plasma drug concentration measurement, which was scheduled 2 h after administration of evocalcet at weeks 16 and 24.

### Statistical analysis

The sample size was determined, based on feasibility, to be 30 as the number of patients on PD from whom information on the efficacy and safety of evocalcet could be obtained. The target sample size of 30 patients allows for a 95% confidence interval (CI) of 31.3–68.7% when the primary endpoint, namely, “the number and percentage of patients who have achieved the mean iPTH level of 60–240 pg/mL during the evaluation period”, is 50% (15 patients), which represents the maximum width of the CI.

Categorical data were summarized as frequency and percentage, and continuous data were summarized in descriptive statistics; i.e., number of patients, mean ± SD, minimum, or median (interquartile range). Interquartile range was calculated as the third quartile (Q3) minus the first quartile (Q1).

The efficacy analysis was carried out using the full analysis set of patients who enrolled in the study and who received evocalcet at least once and had iPTH levels measured at least once after the study began. In addition, an ad-hoc analysis was performed on levels of intact FGF23 and other biochemical markers using the Wilcoxon signed-rank test and a *t* test. The safety analysis was carried out using the safety analysis set, which included patients enrolled in the study, and who were administered evocalcet at least once.

## Results

### Patient background

Written informed consent was obtained from 52 PD patients with SHPT. Of these, 10 were judged to be ineligible for enrollment, and a total of 42 patients were subsequently enrolled. Three patients were withdrawn from the study before starting treatment due to one instance each of eligibility violation, physician decision, and patient withdrawal. The demographics of all patients who began the study (*n* = 39) are presented in Table 1.

**Table 1:** Patient demographics

	*n* = 39
Sex, male	23 (59.0)
Age, years	62.4 ± 10.1
≥ 65 years	18 (46.2)
Body weight, kg	65.1 ± 11.9
Body mass index, kg/m^2^	24.9 ± 3.2
Duration of dialysis, months	32.4 ± 23.6
Dialysate calcium concentration	
2.3 mEq/L	4 (10.3)
2.5 mEq/L	10 (25.6)
3.5 mEq/L	15 (38.5)
Other	10 (25.6)
Treatment history	
Use of cinacalcet before screening	9 (23.1)
Use of vitamin D receptor activators at week 0	25 (64.1)
Use of phosphate binder/calcium preparation at week 0	31 (79.5)
Primary disease	
Diabetic nephropathy	9 (23.1)
Chronic glomerulonephritis	15 (38.5)
Nephrosclerosis	6 (15.4)
Polycystic kidney disease	5 (12.8)
Other	4 (10.3)
Complications	
Diabetes	12 (30.8)
Congestive heart failure	1 (2.6)
Type of dialysis	
CAPD	29 (74.4)
APD	10 (25.6)

Data are shown as *n* (%) or mean ± SD

*APD* automated peritoneal dialysis, *CAPD* continuous ambulatory peritoneal dialysis, *SD* standard deviation

**Fig. 2:**
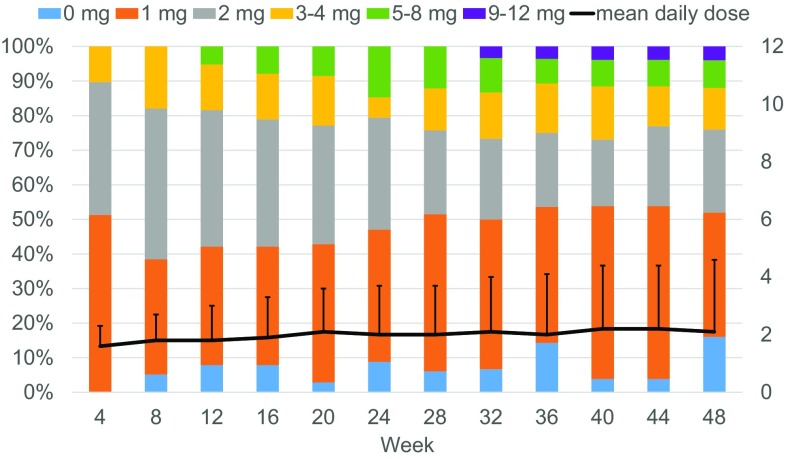
Time course of evocalcet dose distribution and mean daily dose during the study

Of the 39 patients, 29 (74.4%) patients were receiving continuous ambulatory peritoneal dialysis (CAPD) and 10 (25.6%) patients were receiving automated peritoneal dialysis (APD). Two patients (5.1%) had a mean daily urine volume < 100 mL. Thirty-nine patients who received evocalcet were included in the safety analysis with a mean ± SD duration of treatment of 43.7 ± 12.5 weeks, and a mean daily dose of 2.3 ± 2.5 mg at week 52. The mean iPTH concentration at baseline was 465.7 ± 281.6 pg/mL, and the mean corrected serum calcium concentration at baseline was 9.13 ± 0.50 mg/dL. In total, 31 patients (79.5%) completed the study up to week 32, and 24 patients (61.5%) completed the study up to week 52 (Fig. 1b). The mean daily dose of evocalcet and the dose distribution over the course of the study are shown in Fig. 2.

### Efficacy analysis

With respect to the primary endpoint, 28 patients (71.8%, 95% CI 55.1–85.0%) achieved mean iPTH concentrations within the range of 60–240 pg/mL during the evaluation period (weeks 30–32), and 20 patients (51.3%) achieved the target iPTH concentration range at week 52 (Table 2). After excluding those patients who did not complete the 52-week study, 83.3% of patients at week 52 (20/24 patients) achieved iPTH levels of 60–240 pg/mL.

**Table 2:** Efficacy results

	Baseline	Evaluation period (Weeks 30–32)	Week 32	Week 52
Patients who achieved the target iPTH concentration^a^, *n* (%)		28 (71.8)	27 (69.2)	20 (51.3)
Change in iPTH concentration from baseline, %	0	−64.4 ± 26.0	−64.6 ± 24.2	−63.5 ± 22.3
Patients who achieved a 30% decrease or more in iPTH level from baseline, *n* (%)		29 (74.4)	28 (71.8)	21 (53.8)
iPTH concentration, pg/mL	465.7 ± 281.6		132.8 ± 62.4^**^	132.0 ± 61.3^**^
Whole PTH concentration, pg/mL	189.8 ± 96.7		63.0 ± 28.7^**^	67.7 ± 32.4^**^
Corrected serum calcium concentration, mg/dL	9.13 ± 0.50		8.71 ± 0.66^**^	8.60 ± 0.53^**^
Ionized calcium concentration, mEq/L	2.28 ± 0.17		2.20 ± 0.17	2.18 ± 0.12^*^
Serum phosphorus concentration, mg/dL	4.86 ± 1.07		4.79 ± 1.40	4.48 ± 1.10
Calcium-phosphorus product, mg/dL	44.55 ± 10.88		41.85 ± 13.29	38.58 ± 9.64^**^
Intact FGF23 concentration, pg/mL	5770 (11,480)		5090 (14,244)	5035 (9672)^*^
Bone turnover markers				
BAP, µg/L	17.57 ± 10.38		12.14 ± 4.44^**^	13.45 ± 4.96^*^
TRACP-5b, mU/dL	646.7 ± 338.3		296.4 ± 134.9^**^	301.2 ± 241.2^**^
Total P1NP, µg/L	282.9 ± 211.3		240.7 ± 242.2	177.5 ± 156.4^**^

Data are shown as *n* (%), mean ± SD, or median (interquartile range), as appropriate. Interquartile range was calculated as the third quartile (Q3) minus the first quartile (Q1)

*BAP* bone-specific alkaline phosphatase, *FGF23* fibroblast growth factor 23, *iPTH* intact parathyroid hormone, *PTH* parathyroid hormone, *P1NP* procollagen type 1 intact N-terminal propeptide, *TRACP-5b* tartrate-resistant acid phosphatase 5b, *SD* standard deviation

^*^P value < 0.05, ^**^P value < 0.01 vs. baseline

^a^Treatment goal recommended by Japanese guidelines: between ≥ 60 pg/mL and ≤ 240 pg/mL

**Fig. 3:**
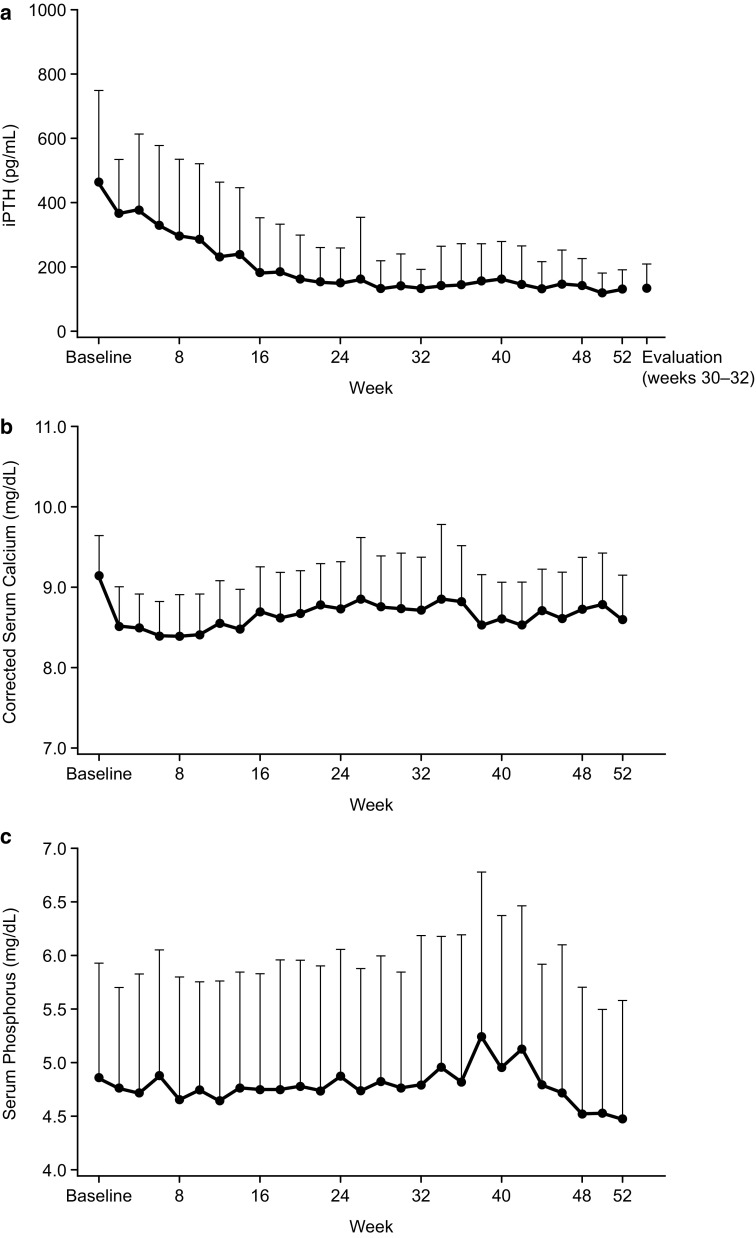
Time-course change in intact parathyroid hormone (iPTH) levels (**a**), corrected serum calcium levels (**b**), and serum phosphorus levels (**c**) during the study. Data are shown as mean ± SD. *SD* standard deviation

Analysis of the secondary efficacy endpoints showed that the mean percent change in iPTH concentration from baseline was − 64.4 ± 26.0% during the evaluation period (weeks 30–32) and − 63.5 ± 22.3% at week 52 (Table 2). The mean iPTH levels were 465.7 ± 281.6 pg/mL at baseline, 132.8 ± 62.4 pg/mL at week 32, and 132.0 ± 61.3 pg/mL at week 52 (Table 2). The time-course change of iPTH concentration during the study is shown in Fig. 3a. In addition, the proportion of patients who achieved ≥ 30% decrease in the concentration of iPTH from baseline during the evaluation period (weeks 30–32) was 29 patients (74.4%, 95% CI 57.9–87.0%) and 21 patients (53.8%) at week 52 (Table 2). After excluding those patients who did not complete the 52-week study, 87.5% of patients (21/24 patients) achieved a ≥ 30% decrease in the concentration of iPTH from baseline at week 52.

The mean whole PTH levels displayed a similar trend, with a baseline level of 189.8 ± 96.7 pg/mL, which decreased to 63.0 ± 28.7 pg/mL at week 32 and was 67.7 ± 32.4 pg/mL at week 52 (Table 2).

The corrected serum calcium levels initially decreased from baseline until week 2 and then remained stable thereafter (Fig. 3b). The mean corrected serum calcium levels were 9.13 ± 0.50 mg/dL at baseline, 8.71 ± 0.66 mg/dL at week 32, and 8.60 ± 0.53 mg/dL at week 52 (Table 2). The mean ionized calcium levels showed minimal differences throughout the study. At baseline, the mean levels of ionized calcium were 2.28 ± 0.17 mEq/L, 2.20 ± 0.17 mEq/L at week 32, and 2.18 ± 0.12 mEq/L at week 52 (Table 2).

Throughout the study, the serum phosphorus levels showed scattered fluctuations (Fig. 3c). However, these levels generally remained constant throughout the study period, showing minimal differences between baseline (4.86 ± 1.07 mg/dL), week 32 (4.79 ± 1.40 mg/dL), and week 52 (4.48 ± 1.10 mg/dL). The median (interquartile range) intact FGF23 level was 5770 (11,480) pg/mL at baseline; this decreased to 5090 (14,244) pg/mL at week 32, and was significantly decreased (*P* < 0.05) to 5035 (9672) pg/mL at week 52 (Table 2).

The concentrations of mean bone turnover markers BAP, TRACP-5b, and total P1NP decreased from 17.57 ± 10.38 µg/L, 646.7 ± 338.3 mU/dL, and 282.9 ± 211.3 µg/L at baseline, respectively, to 12.14 ± 4.44 µg/L, 296.4 ± 134.9 mU/dL, and 240.7 ± 242.2 µg/L at week 32, respectively (Table 2). Both BAP and TRACP-5b levels remained stable after week 32 and were 13.45 ± 4.96 µg/L and 301.2 ± 241.2 mU/dL, respectively, at week 52. In contrast, total P1NP levels continued to decrease and were 177.5 ± 156.4 µg/L at week 52. There were no significant differences in efficacy after subgroup analyses of patients stratified by type of dialysis, residual renal function, history of prior cinacalcet treatment, and starting dose of evocalcet (data not shown).

### Safety analysis

AEs were observed in 39 patients (100%). Corrected serum calcium decreased in seven patients (17.9%), and hypertension occurred in seven patients (17.9%) (Table 3). Adverse drug reactions were observed in 18 patients (46.2%). AEs related to decrease in serum calcium concentrations (coded as MedDRA Preferred Terms “corrected calcium decreased” and “blood calcium decreased”), which occurred in nine patients (23.1%), were the most common (Table 3).

**Table 3:** Adverse events occurring in ≥ 5% of patients

	Adverse events (*n* = 39)	Drug-related adverse events (*n* = 39)
Nasopharyngitis	17 (43.6)	0 (0.0)
Catheter site infection	11 (28.2)	0 (0.0)
Corrected calcium decreased	7 (17.9)	7 (17.9)
Hypertension	7 (17.9)	0 (0.0)
Diarrhea	4 (10.3)	0 (0.0)
Peritonitis	4 (10.3)	0 (0.0)
Contusion	4 (10.3)	0 (0.0)
Iron deficiency anemia	3 (7.7)	1 (2.6)
Vomiting	3 (7.7)	1 (2.6)
Pruritus	3 (7.7)	1 (2.6)
Gastroenteritis	3 (7.7)	0 (0.0)
Hyperphosphatemia	3 (7.7)	0 (0.0)
Rash	3 (7.7)	0 (0.0)
Blood calcium decreased	2 (5.1)	2 (5.1)
Myocardial ischemia	2 (5.1)	1 (2.6)
Pyrexia	2 (5.1)	1 (2.6)
Decreased appetite	2 (5.1)	1 (2.6)
Angina pectoris	2 (5.1)	0 (0.0)
Conjunctival hemorrhage	2 (5.1)	0 (0.0)
Edema	2 (5.1)	0 (0.0)
Bronchitis	2 (5.1)	0 (0.0)
Conjunctivitis	2 (5.1)	0 (0.0)
Folliculitis	2 (5.1)	0 (0.0)
Pharyngitis	2 (5.1)	0 (0.0)
Rhinitis	2 (5.1)	0 (0.0)
Blood creatine phosphokinase increased	2 (5.1)	0 (0.0)
Dehydration	2 (5.1)	0 (0.0)
Hyperkalemia	2 (5.1)	0 (0.0)
Hypokalemia	2 (5.1)	0 (0.0)
Back pain	2 (5.1)	0 (0.0)
Muscle spasms	2 (5.1)	0 (0.0)
Osteoarthritis	2 (5.1)	0 (0.0)
Insomnia	2 (5.1)	0 (0.0)
Sleep apnea syndrome	2 (5.1)	0 (0.0)
Upper respiratory tract inflammation	2 (5.1)	0 (0.0)
Dermatitis	2 (5.1)	0 (0.0)

Adverse events are presented as MedDRA Preferred Terms. Data are shown as *n* (%)

AEs that were classified as serious occurred in 16 patients (41.0%). Serious AEs not considered related to the study drug occurring in ≥ 5% of patients included peritonitis (four patients, 10.3%), catheter site infection (three patients, 7.7%), and angina pectoris (two patients, 5.1%). Severe AEs included one subject experiencing an altered state of consciousness, which resulted in death; however, a causal relationship with evocalcet was ruled out. Regarding the incidence of GI-related AEs (nausea, vomiting, abdominal discomfort, abdominal distension, and decreased appetite), vomiting occurred in only three patients (7.7%). Vomiting was classified as a drug-related AE in one patient (2.6%). No other measure (12-lead ECG, laboratory parameters, body weight, or vital signs) showed clinically significant or specific trends.

## Discussion

This was the first study evaluating the long-term efficacy and safety of evocalcet in Japanese PD patients with SHPT. The results show that evocalcet is an efficacious treatment in Japanese PD patients with SHPT, as observed by the high proportion of patients (71.8%, 95% CI 55.1–85.0%) who achieved a mean iPTH level of 60–240 pg/mL during the evaluation period (weeks 30–32). In a recently published study, a high proportion of patients undergoing HD also achieved the target iPTH concentration [6]; therefore, evocalcet is considered to be equally efficacious for both HD and PD.

When evaluating the proportion of patients who achieved the target iPTH concentration at week 32 and at the end of this study (week 52), there was a reduction in the proportion of patients achieving the target iPTH concentration range. This is related to the inclusion of patients that withdrew from the study. When excluding those who discontinued treatment, the proportion of patients achieving the target iPTH concentration range remained high at 83.3%. Subgroup analysis showed that the efficacy of evocalcet was not limited by patient demographics such as dialysis type, residual renal function, or history of prior cinacalcet treatment.

Phosphorus control is important for PD patients as well as HD patients given its association with an increased risk of death [11]. Therefore, the efficacy of evocalcet is supported by the stable phosphorus levels observed in this study. These stable calcium and phosphorus levels were controlled through the appropriate dose-adjustment of not only evocalcet, but also of other relevant concomitant drugs.

In a previous report, intact FGF23 levels decreased significantly with cinacalcet treatment [12]. In the present study, intact FGF23 decreased over time from baseline to week 52; however, this reduction was only minor, owing to the small number of patients included in the study and the large variation in intact FGF23 levels at baseline. In addition, a head-to-head phase III study in SHPT patients receiving HD also showed that intact FGF23 decreased by a similar amount in both evocalcet and cinacalcet groups when the dose or dosing regimen of activated vitamin D drug or its derivative were not changed [6]. These results indicate that evocalcet treatment may reduce intact FGF23 levels independent of activated vitamin D, as was similarly shown with cinacalcet treatment.

In this study, the dose of evocalcet was adjusted to approximately 1–2 mg for most patients, as was also reported in our long-term phase III study in SHPT patients receiving HD [13]. Although the dose in one patient was increased to the maximum allowed dose of 12 mg, evocalcet was still well tolerated and no new safety concerns arose. The safety of evocalcet administration in PD patients was also shown by the absence of any severe adverse drug reactions. Evocalcet was also shown to be tolerable for patients with SHPT as evidenced by the absence of significant GI-related side effects as are commonly observed with the administration of calcimimetic agents. This is supported by a recent double-dummy, double-blind design study comparing evocalcet and cinacalcet [6], showing that the overall incidence of GI-related AEs was lower in the evocalcet than in the cinacalcet group (59 patients [18.6%; 95% CI 14.5–23.3%] vs. 104 patients [32.8%; 95% CI 27.7–38.3%]).

Evocalcet has been developed as an oral drug and represents the possibility of being a superior alternative for the treatment of SHPT in PD patients [5–8, 13–16]. Although oral cinacalcet is reported to be an effective treatment for SHPT, the high frequency of GI-related AEs can cause poor adherence rates and drug discontinuation, thus preventing adequate treatment [17]. The low incidence of GI-related AEs reported in this study shows that evocalcet may be more tolerable and therefore more efficacious than other currently available therapies. In the context of calcimimetic drugs other than cinacalcet, the injectable therapy etelcalcetide is not currently approved for patients undergoing PD [18–21].

The results of this study should be considered in the context of its limitations—namely, the small number of patients enrolled, which was largely due to the low number of patients currently receiving PD in Japan [3]. This was also a single-arm study, and therefore, the superiority of evocalcet over cinacalcet could not be determined in SHPT patients receiving PD. Furthermore, as active vitamin D and its derivatives were permitted, a possible confounding effect from concomitant vitamin D cannot be ruled out.

## Conclusion

In conclusion, this single-arm, open-label, dose-adjustment study of evocalcet administered once daily for 32 weeks, followed by an extension treatment for 20 weeks, showed that SHPT patients receiving PD achieved the target mean iPTH levels of 60–240 pg/mL. In addition, corrected serum calcium and serum phosphorus levels remained constant until week 52 with evocalcet administration. No new safety concerns, including GI-related AEs, were observed, and no severe adverse drug reactions were reported. Therefore, evocalcet can be considered a more tolerable option for SHPT patients and will contribute to enhancing treatment adherence and reducing discontinuation rates.

## Electronic supplementary material

Below is the link to the electronic supplementary material.

Supplementary material 1 (PDF 9 KB)
