# Supplementary material for: Efficacy and safety of evocalcet in Japanese peritoneal dialysis patients
Source: Clin Exp Nephrol. 2019 Apr 6;23(6):739–48. doi: 10.1007/s10157-019-01692-y (PMC6586709; doi:10.1007/s10157-019-01692-y)
Supplement: Supplementary file 1 — Supplementary material 1 (PDF 9 KB) [file 10157_2019_1692_MOESM1_ESM.pdf]

Supplementary Table S1: List of participating centers

Principal Investigator	Institution
Yoshimitsu Hayashi	Fukushima Medical University Hospital
Hidetomo Nakamoto	Saitama Medical University Hospital
Shoji Koga	Edogawa Hospital
Shoji Koga	Medical Plaza Edogawa
Ichiro Okido	The Jikei University Hospital
Minoru Kubota	Ouji Hospital
Fumihiko Koiwa	Showa University Fujigaoka Hospital
Masahiro Takeda	Komatsu Municipal Hospital
Terumasa Hayashi	Osaka General Medical Center
Makoto Hiramatsu	Okayama Saiseikai Outpatient Center Hospital
Hideki Kawanishi	Tsuchiya General Hospital
Hidetoshi Kanai	Kokura Memorial Hospital
Sakuya Ito	Kurume University Hospital
Kazuhiko Tsuruya	Kyushu University Hospital
Koji Mitsuiki	Fukuoka Red Cross Hospital
Hirofumi Ikeda	Munakata Medical Association Hospital
